# Seminal Bacterioflora of Two Rooster Lines: Characterization, Antibiotic Resistance Patterns and Possible Impact on Semen Quality

**DOI:** 10.3390/antibiotics12020336

**Published:** 2023-02-05

**Authors:** Eva Tvrdá, Michaela Petrovičová, Filip Benko, Michal Ďuračka, Ján Kováč, Tomáš Slanina, Lucia Galovičová, Jana Žiarovská, Miroslava Kačániová

**Affiliations:** 1 Institute of Biotechnology, Faculty of Biotechnology and Food Sciences, Slovak University of Agriculture in Nitra, Tr. A. Hlinku 2, 94976 Nitra, Slovakia; 2 Institute of Applied Biology, Faculty of Biotechnology and Food Sciences, Slovak University of Agriculture in Nitra, Tr. A. Hlinku 2, 94976 Nitra, Slovakia; 3 Department of Neuroscience, Second Faculty of Medicine (2. LF UK), V Úvalu 84, 15006 Prague, Czech Republic; 4 AgroBioTech Research Centre, Slovak University of Agriculture in Nitra, Tr. A. Hlinku 2, 94976 Nitra, Slovakia; 5 Department of Fruit Science, Viticulture and Enology, Faculty of Horticulture and Landscape Engineering, Slovak University of Agriculture, Tr. A. Hlinku 2, 94976 Nitra, Slovakia; 6 Institute of Plant and Environmental Sciences, Faculty of Agrobiology and Food Resources, Slovak University of Agriculture, Tr. A. Hlinku 2, 94976 Nitra, Slovakia; 7 Department of Bioenergetics, Food Analysis and Microbiology, Institute of Food Technology and Nutrition, University of Rzeszow, Cwiklinskiej 1, 35-601 Rzeszow, Poland

**Keywords:** roosters, bacteriospermia, semen, antibiotic resistance, Lohmann Brown, ROSS 308

## Abstract

This study aimed to characterize the bacterial profiles and their association with selected semen quality traits among two chicken breeds. Thirty Lohmann Brown and thirty ROSS 308 roosters were selected for semen quality estimation, including sperm motility, membrane and acrosome integrity, mitochondrial activity, and DNA fragmentation. The oxidative profile of the semen, including the production of reactive oxygen species (ROS), antioxidant capacity, protein, and lipid oxidation, were assessed as well. Moreover, the levels of pro-inflammatory cytokines, including tumor necrosis factor alpha (TNF-α), interleukins 1 and 6 (IL-1, IL-6) and C-reactive protein, as well as the concentrations of selected antibacterial proteins (cathelicidin, β-defensin and lysozyme) in the seminal plasma were evaluated with the enzyme-linked immunosorbent assay. The prevailing bacterial genera identified by the matrix-assisted laser desorption/ionization time-of-flight mass spectrometry were *Citrobacter* spp., *Enterococcus* spp., *Escherichia* spp. and *Staphylococcus* spp. While the bacterial load was significantly higher in the ROSS 308 line (*p* < 0.05), a higher number of potentially uropathogenic bacteria was found in the Lohmann Brown roosters. Antimicrobial susceptibility tests revealed a substantial resistance of randomly selected bacterial strains, particularly to ampicillin, tetracycline, chloramphenicol, and tobramycin. Furthermore, Lohmann Brown ejaculates containing an increased proportion of *Escherichia coli* presented with significantly (*p* < 0.05) elevated levels of TNF-α and IL-6, as well as ROS overproduction and lipid peroxidation. Inversely, significantly (*p* < 0.05) higher levels of β-defensin and lysozyme were found in the semen collected from the ROSS 308 roosters, which was characterized by a higher quality in comparison to the Lohmann Brown roosters. In conclusion, we emphasize the criticality of bacteriospermia in the poultry industry and highlight the need to include a more complex microbiological screening of semen samples designated for artificial insemination.

## 1. Introduction

Due to a short generation interval and higher feed conversion efficiency, poultry represents a cost-effective source of animal protein that is nutritious, palatable and easily digestible [1]. Economically speaking, the poultry industry presents job opportunities, a high chance for investment and a source of income for smallholders worldwide [2]. Consequently, the demand for poultry products faces a rising tendency, with a further 17% increase predicted by 2027 [3]. Hence, there is a critical need to increase the production of chicken coops, which is limited by the ability of the animals to reproduce [4].

Fertility is the primary requirement of poultry farming, as the number of fertilized eggs destined for hatching determines the final profitability of the production [4]. Even though both males and females contribute to the fertilization rate, male fertility may be easily impacted by a wide variety of both intrinsic and extrinsic factors, such as age, feed regime and health status. In addition, overbreeding in favor of muscle gain, particularly in broiler chicken, complicates the natural mating process. As such, artificial insemination (AI) has become a fundamental tool for poultry production [1]. This process allows a more effective utilization of males with valuable genetic traits and a desirable reproductive performance, leading to high hatchability, which is impossible to accomplish under natural mating conditions [1,2]. Furthermore, AI implementation will save production costs by decreasing the number of stud roosters, thus saving expenses for maintenance, feed, and operation [1]. Nevertheless, as discussed earlier, the success of AI is principally dependent upon the quality of ejaculates needed to maximize the reproductive output, while reducing the wastage of a rooster’s investment in producing spermatozoa [3].

In the past, semen collected from clinically healthy males was considered free from bacteria, which has resulted in the male reproductive microbiome not being well described [5]. However, recent studies have found that bacteriocenoses are a normal part of the male urogenital system and bodily fluids, including urine and semen [6,7,8,9]. As 16S ribosomal RNA sequencing and matrix-assisted laser desorption/ionization-time of flight (MALDI-TOF) mass spectrometry (MS) have been applied to study the animal microbiome, associations between the bacterial profiles of semen and sperm quality have been gradually revealed [6,7,8,9,10]. This is particularly true in poultry, where the proximity of the gastrointestinal and reproductive system predisposes ejaculates to be easily contaminated by bacteria [8,11]. The deterioration of the sperm plasma membrane and DNA, the occurrence of pathological alterations to spermatozoa, oxidative stress and apoptosis, as well as sperm agglutination and immobilization leading to a decreased semen quality, have been previously associated with bacteriospermia in bulls [6,9], stallions [10], rabbits [12], boars [13] and turkeys [8]. If a contained semen sample is used for AI, bacteria may be easily transferred to the female [14], potentially causing urogenital infections, which are responsible for a decreased laying frequency and hatchability [15,16]. Furthermore, bacteria may be easily transmitted to eggs and meat, posing a potential threat to the consumer’s health [17,18]. The spread of pathogenic bacteria in poultry production is also a major contributor to the increased morbidity and mortality of animals, resulting in an estimated annual economic loss of more than two- billion US dollars [19,20,21].

While different strategies have been developed to prevent or counteract bacterial transmission in poultry production [22,23], antibiotics are traditionally a preferred option for disease control because of their affordability and availability. Nevertheless, the evidence gathered from recent studies emphasizes the occurrence of *Escherichia coli*, enterococci, staphylococci, or *Campylobacter* found in poultry or poultry products that present with a substantial resistance to the antibiotics routinely used in animal production, such as vancomycin, streptomycin, chloramphenicol, tetracycline, erythromycin or ampicillin [24,25,26,27,28]. Such horizontal or vertical transmission of bacterial drug resistance has become a serious threat to public health and to the stability of the food chain and ecosystems [20]. It is primarily for this reason that the use of “growth-promoting” antibiotics in feed was entirely banned in 2006, and their use as supplements in poultry semen extenders is strictly regulated [20,29].

While studies focused on reproduction in roosters have primarily assessed changes in the sperm quality affected by age [30], nutrition [31], season [32] or genotype [33], knowledge of the impact of the bacteriome on rooster semen characteristics is very sparse, partially because the latest major studies employing traditional bacteriological techniques are between ten and twenty years ago [34,35,36]. As such, this study was designed to characterize the bacteriome of rooster semen using advanced and highly accurate MALDI-TOF MS, which has been successfully used to study the bacterial profiles of bull [6], ram [7], turkey [8], stallion [10], rabbit [12] and boar [13] ejaculates. All identified bacteria were then subjected to comprehensive antimicrobial susceptibility testing against an array of antibiotics, most of which are routinely used in animal andrology [29]. Furthermore, we studied any possible fluctuations of the selected inflammatory molecules that may play a role in the immune response to bacterial presence in semen, as well as the proteins that may contribute to the antibacterial protection of spermatozoa. In this sense, instead of a global description of the above-mentioned conventional and molecular semen quality traits, we chose to follow a comparative approach, by studying semen samples from two different chicken breeds representing a layer type (egg laying Lohmann Brown breed) and a broiler type (ROSS 308 breed raised for meat production).

## 2. Results

### 2.1. Identification of Bacteria

**Figure 1:**
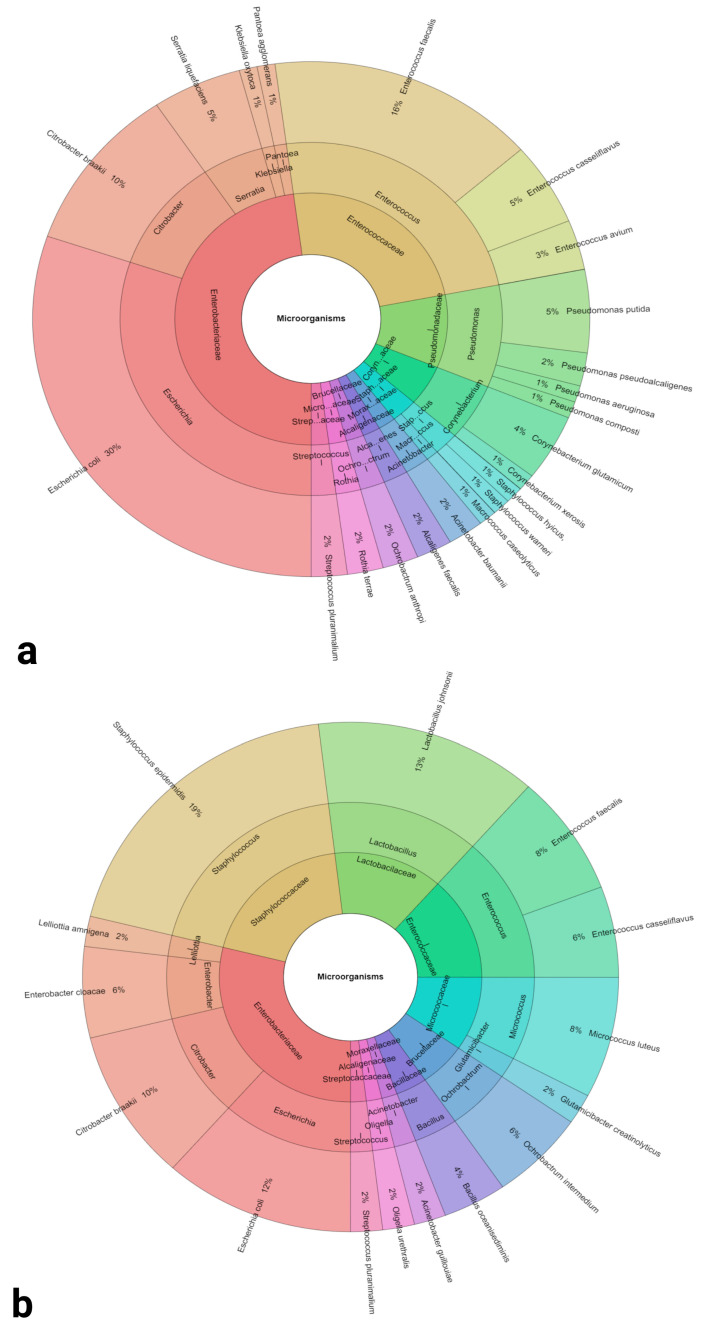
Krona charts of the bacteria identified in semen collected from Lohmann Brown (**a**) and ROSS 308 (**b**) roosters. Outer ring: species, middle ring: genus, inner ring: family.

Matrix assisted laser desorption/ionization time-of-flight mass spectrometry (MALDI TOF MS) was used for the identification of bacteria isolated from semen samples collected from 2 breeds of roosters. In the case of the Lohmann Brown roosters, 10 families, 15 genera and, overall, 27 species were retrieved, with *Escherichia coli* (30%), *Enterococcus faecalis* (16%) and *Citrobacter braaki* (10%) being the predominant species. G^−^ bacteria were more frequent (62%) in comparison to G^+^ bacteria (38%). In the meantime, 10 families, 14 genera and 16 species were identified in the ejaculates obtained from the ROSS 308 roosters. In this case, *Staphylococcus epidermidis* (19%), *Lactobacillus johnsonii* (13%) and *Escherichia coli* (12%) were the most prevalent species present in the samples; most of the bacterial species were represented by G^+^ bacteria (62%) as opposed to G^−^ bacteria (38%). A summary of all species retrieved from the semen of both rooster breeds are provided by Table 1 and Figure 1. With respect to the bacterial load, a higher quantity of bacteria was recorded in the ROSS 308 breed than the Lohmann Brown breed (*p* = 0.0083; Table 1).

### 2.2. Biodiversity Assessment

**Figure 2:**
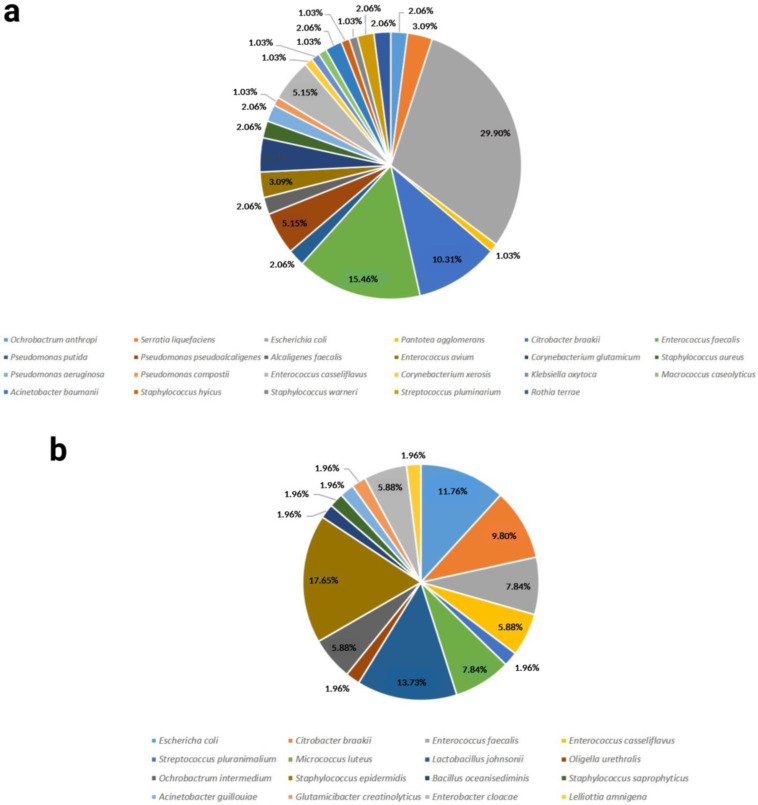
Bacterial species identified in the analyzed breeds and their proportions. (**a**) Lohman Brown breed, (**b**) ROSS 308 breed.

A total of 27 different bacterial species were found in the samples from the Lohman Brown breed, compared to 16 species in the ROSS 308 breed. The most abundant species was *Escherichia coli* in the Lohman Brown roosters, with a proportion of 29.90%, and *Staphylococcus epidermis* in the ROSS 308 roosters, with a proportion of 17.65% (Figure 2).

**Figure 3:**
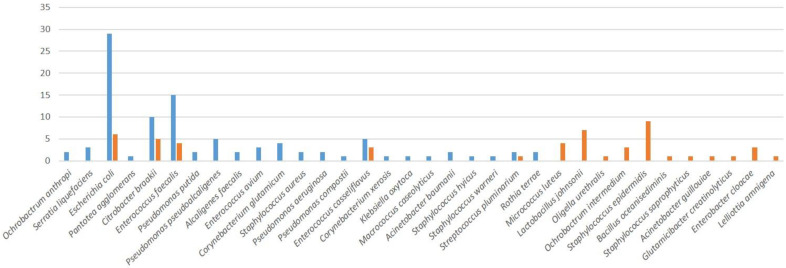
Distribution of identified bacterial species between the analyzed breeds.

Only five bacterial species were found in both breeds; most of the species present were breed-specific (Figure 3).

Based on the obtained diversity indices, the richness of the bacterial species was found to be higher in the Lohman Brown breed. The calculated diversity indices were minimal in their values for both analyzed breeds, which is strongly required in terms of reproductive biology. The Berger-Parker Index values were also low in both groups, which corresponds to a small domination of individual bacterial species throughout the analyzed samples. The values of the indexes were affected by a small abundance and numbers of bacteria that were present in the ejaculates (Table 2).

### 2.3. Bacterial Resistence

Randomly selected isolates from each species identified in both groups were subjected to the assessment of their antimicrobial resistance (Table 3) against ampicillin, chloramphenicol, gentamycin, imipenem, levofloxacin, tetracycline, tigecycline and tobramycin. Any inhibition zones were evaluated following the European Committee on Antimicrobial Susceptibility Testing (EUCAST) instructions. While sensitivity towards all of the antibiotics was observed in the cases of *Glutamicibacter creatinolyticus*, *Macrococcus caseolyticus, Oligella urethralis*, *Pseudomonas composti*, *Pseudomonas pseudoalcaligenes* and *Streptococcus pluranimalium*, resistance patterns were observed across all of the bacterial species evaluated in this study. Numerous isolates were revealed to have intermediate to full resistance against ampicillin, particularly in the cases of *Citrobacter braakii*, enterococci, *Escherichia coli* or *Staphylococcus epidermidis*. Resistance to tetracycline was observed in several *Staphylococcus epidermidis* isolates, while a few *Escherichia coli* isolates were resistant to chloramphenicol. Multiresistance patterns against several antibiotics were recorded, particularly in the cases of *Citrobacter braakii*, *Enterococcus faecalis*, *Escherichia coli* and *Staphylococcus epidermidis*.

### 2.4. Semen Quality Parameters

**Figure 4:**
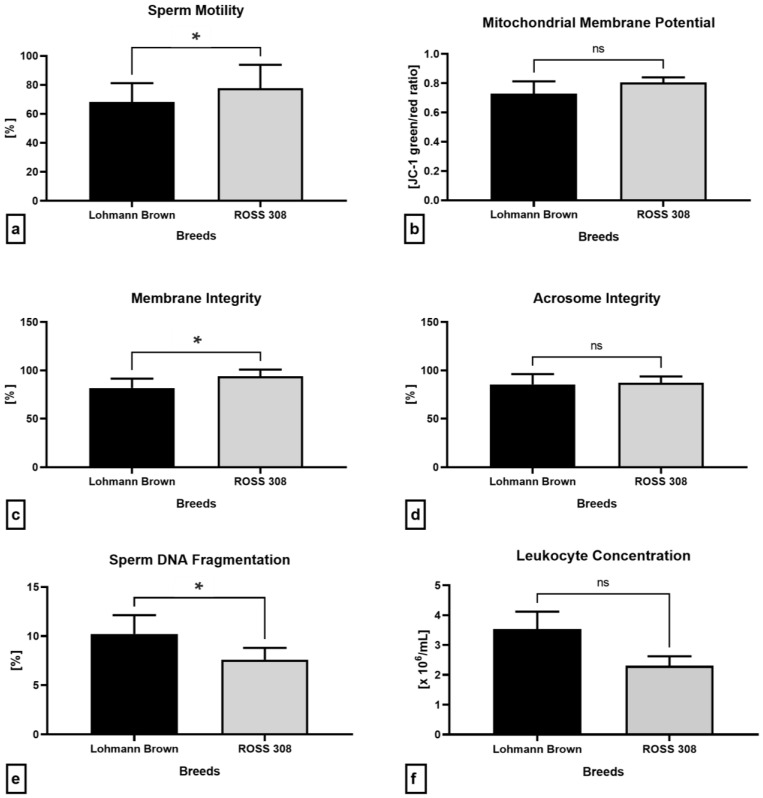
Conventional semen quality parameters including the sperm motility (**a**), mitochondrial membrane potential (**b**), membrane integrity (**c**), acrosome integrity (**d**), DNA integrity (**e**) and leukocyte concentration (**f**) of samples collected from Lohmann Brown and ROSS 308 roosters. Mean ± SD. Significant (*) if *p* < 0.05. ns—non-significant.

Figure 4a reveals a significantly lower sperm motility in the samples collected from the Lohmann Brown roosters in comparison to the ROSS 308 (*p* = 0.0437). While no significant differences among the breeds were recorded in the cases of the mitochondrial activity (*p* = 0.0931; Figure 4b) and acrosome integrity (*p* = 0.8702; Figure 4d), the spermatozoa collected from the ROSS 308 roosters presented with a significantly higher membrane stability (*p* = 0.0003; Figure 4c) and DNA integrity (*p* = 0.0321; Figure 4e) when compared to the Lohmann Brown breed. In the meantime, higher, although non-significant, amounts of leukocytes were recorded in the Lohmann Brown ejaculates in comparison to the BOSS 308 breed (*p* = 0.1475; Figure 4f).

### 2.5. Oxidative Profile

**Figure 5:**
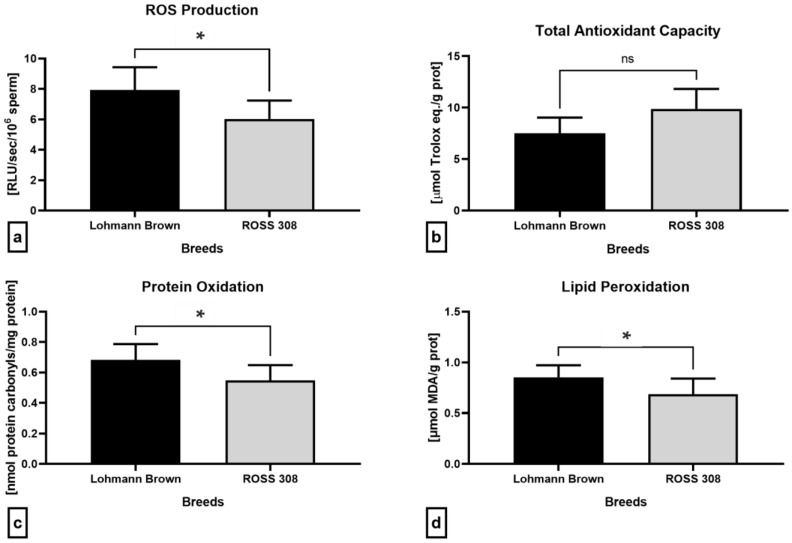
Oxidative characteristics of semen samples collected from Lohmann Brown and ROSS 308 roosters, represented by the production of reactive oxygen species (**a**), total antioxidant capacity (**b**), protein oxidation (**c**) and lipid peroxidation (**d**). Mean ± SD. Significant (*) if *p* < 0.05. ns—non-significant.

Luminol-based luminescent analysis revealed that the ejaculates collected from the Lohmann Brown roosters presented with significantly higher ROS levels (*p* = 0.0240; Figure 5a) in comparison with the ROSS 308 roosters, correspondingly to significantly higher amount of protein carbonyls (*p* = 0.0217; Figure 5b) and malondialdehyde (*p* = 0.0455; Figure 5d). Inversely, the seminal plasma obtained from the ROSS 308 roosters had a higher total antioxidant capacity, although no significant differences were observed (*p* = 0.4286; Figure 5b).

### 2.6. Immunological Profile of Semen

**Figure 6:**
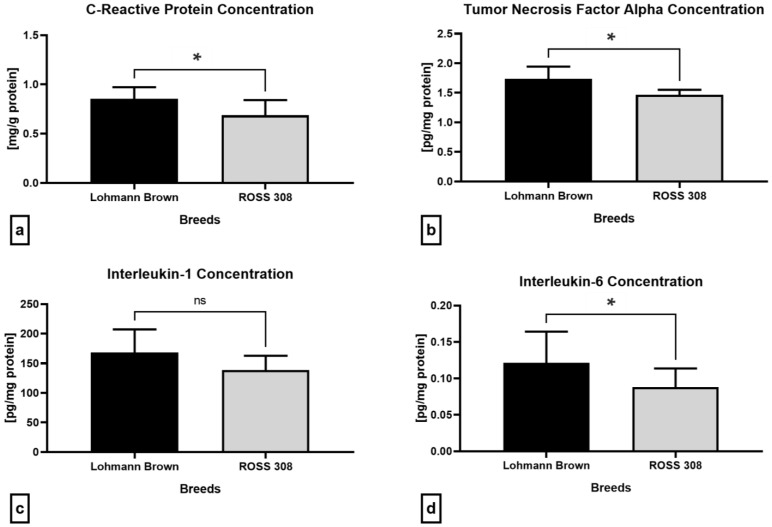
Immunological profile of semen samples collected from Lohmann Brown and ROSS 308 roosters, represented by the C-reactive protein (**a**), tumor necrosis factor alpha (**b**), interleukin-1 (**c**) and interleukin-6 (**d**). Mean ± SD. Significant (*) if *p* < 0.05. ns—non-significant.

The quantification of the selected pro-inflammatory molecules carried out by an enzyme-linked immunosorbent assay (ELISA) revealed significantly higher concentrations of C-reactive protein (CRP; *p* = 0.0455), tumor necrosis factor alpha (TNF-α; *p* = 0.0330) and interleukin-6 (IL-6; *p* = 0.0415) in the seminal plasma of the Lohmann Brown roosters than the ROSS 308 breed (Figure 6a,b,d). Similarly, differences among the breeds were found in the levels of interleukin-1 (IL-1), although without statistical significance (*p* > 0.9999; Figure 6c).

### 2.7. Antibacterial Proteins

**Figure 7:**
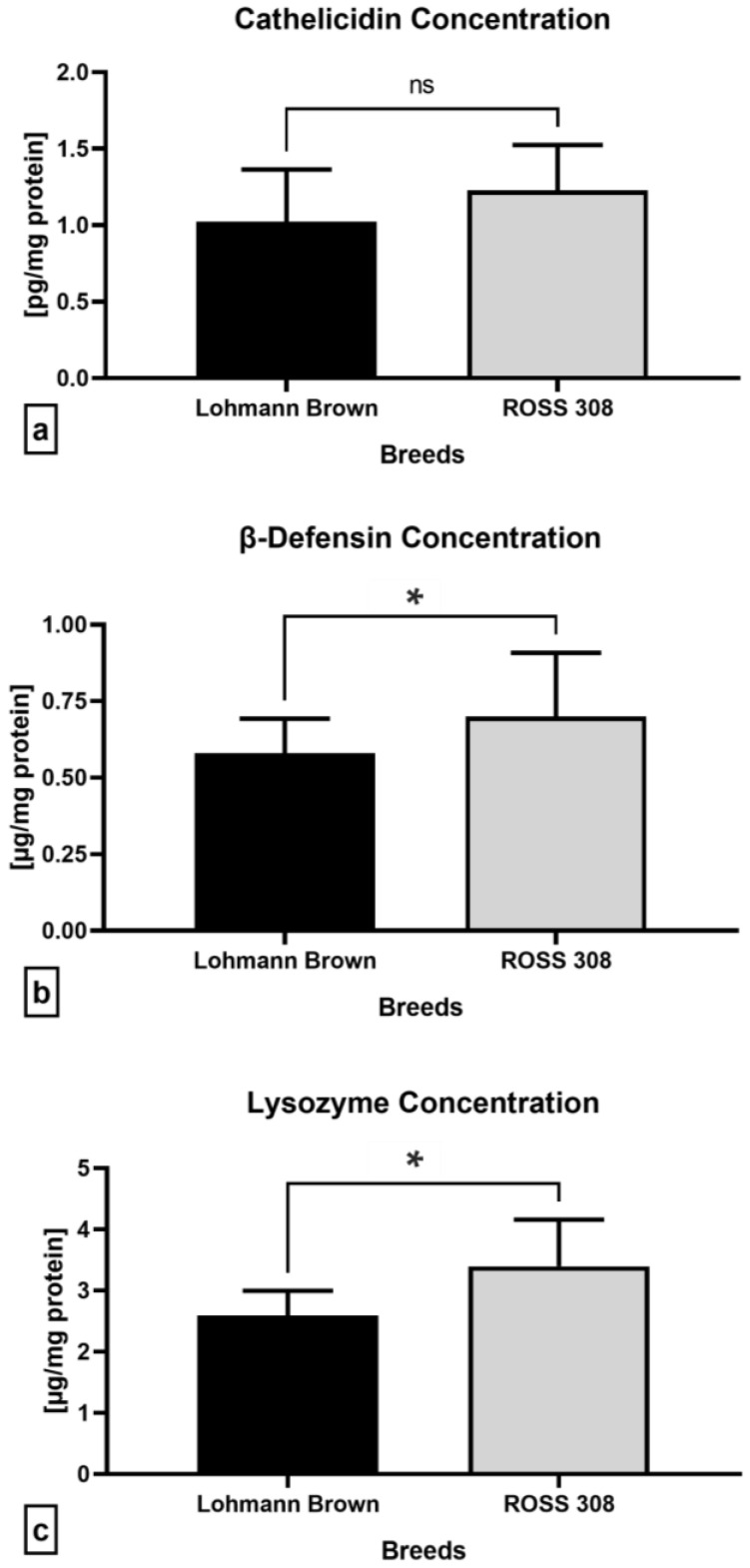
Levels of cathelicidin (**a**), β-defensin (**b**) and lysozyme (**c**) in ejaculates collected from Lohmann Brown and ROSS 308 roosters. Mean ± SD. Significant (*) if *p* < 0.05. ns—non-significant.

With respect to the assessment of the selected proteins with antibacterial properties, significantly higher concentrations of β-defensin (*p* = 0.0373; Figure 7b) and lysozyme (*p* = 0.0266; Figure 7c) were recorded in the ejaculates collected from the ROSS 308 roosters than the Lohmann Brown line. Correspondingly, the ROSS 308 semen samples presented with a higher cathelicidin concentration compared to the Lohmann Brown roosters, although no statistical significance was detected (*p* = 0.3193; Figure 7a).

## 3. Discussion

Since the very first reports describing the presence of bacteria in animal semen [37] and their effects on male gametes [38], published in the 1940s, over 7000 original studies on bacteriospermia have emerged to date. Among these, more than 40% have been published in the last ten years, confirming the rising importance of this topic across the scientific, medical and veterinary communities.

It is now known that, depending on the collection protocol and type of bacteriological analysis, most semen samples collected, even from healthy stud males, are contaminated with bacteria [6,7,8,9]. This is particularly evident in avian species, whose inherent anatomical peculiarities predispose their semen to often contain potentially uropathogenic bacteria [8,34,39,40]. Interestingly, an up-to-date characterization of the seminal bacteriome in breeding roosters by taking advantage of modern identification techniques is still missing. Furthermore, an understanding of the bacterial action within a broader context of the changes to conventional, as well as non-conventional, semen quality traits could assist in the development of novel strategies to manage bacteriospermia in the poultry industry.

As suggested by earlier studies, the severity of the effects bacteria may exhibit on the sperm quality depends upon the bacterial diversity and overall quantity of bacteria present in the sample, also known as the bacterial load [6,8]. With respect to bacterial load, a significantly higher bacterial quantity was found in the ejaculates collected from the ROSS 308 broilers. This may be explained by the size of the animals. Broiler breeds are larger, heavier, and more pressure is required to successfully collect the ejaculate. Consequently, this may lead to higher amounts of bacteria passing through the vas deferens with the seminal fluid [41]. At the same time, it may be plausible to speculate that in any highly specialized animal line or breed subjected to greater selection pressure, an additional burden is placed on the immune system, which is inherently designed to detect and eliminate any potential pathogen. In this sense, the increase in the antibacterial proteins found in the seminal plasma may directly correspond to a higher bacterial load in the semen, and thus indicates a broader response of the local immunity towards the presence of a higher concentration of bacteria. Nevertheless, to confirm this hypothesis on a systemic level, the quantification of the inflammatory factors and antibacterial proteins should be performed in the blood plasma.

On the other hand, notable differences were observed in the bacterial diversity among the breeds. Genera such as *Escherichia* spp., *Staphylococcus* spp., *Citrobacter* spp. and *Enterococcus* spp. were identified in both breeds, which corresponds to the earlier bacteriological studies, methodically based on traditional selective growth media, Gram staining and biochemical assays [39,40]. Nevertheless, a greater prevalence of *Escherichia coli*, alongside the occurrence of *Pseudomonas* spp. and *Corynebacterium* spp., as well as typical uropathogens including *Acinetobacter baumannii* and *Staphylococcus aureus*, was observed in the Lohmann Brown ejaculates. In the meantime, *Staphylococcus epidermidis* was the predominant bacterium identified in the ROSS 308 roosters, accompanied by a relatively high prevalence of *Lactobacillus johnsonii*, as well as the presence of rare bacterial species such as *Ochrobactrum intermedium*, *Oligella urethralis*, *Glutamicibacter creatinolyticus* or *Lelliottia amnigena*. These notable differences are subject to speculation as both groups of roosters were of similar age and kept under identical conditions. Nevertheless, these variations might have been caused by a slightly different metabolism between both chicken types, which may be associated with differences in the shape, function, and microbiome of the gastrointestinal tract [42]. Very little information is available with respect to the unusual bacterial species found in the semen of both breeds; thus, their origin in the semen and possible impact on the resulting sperm quality will be subject to further research.

It seems plausible to speculate that, in this study, the semen quality was affected more by the bacterial diversity and prevalence than the bacterial load, as lower values of the conventional sperm quality parameters were found in the Lohmann Brown ejaculates, which contained more potentially uropathogenic bacteria, in particular *Escherichia coli*. On the other hand, *Lactobacillus* spp., found in the ROSS 308 semen specimens, has been reported to exhibit beneficial effects on the digestive tract of poultry, and may be used as probiotic supplement, with a subsequent positive impact on the sperm production and hatchability [43]. Furthermore, a recent study has revealed the stimulating in vitro effects of selected lactobacilli on the sperm motility, mitochondrial activity and antioxidant characteristics during a short-term co-incubation [44].

As suggested by Zhang et al. [45], bacterial adhesion to the sperm surface as the first event of bacterial contamination may result in an increased load of cells and thus impair the membrane integrity of the spermatozoa. *Escherichia coli* contains polymeric structures, called “fimbriae”, that serve to establish an attachment of the bacterium to the sperm head [46] and tail [47]. The subsequent sperm-bacterial interactions may result in sperm agglutination and initiate biofilm formation [48], which will result in sperm immobilization and the deterioration of the membranous structures of male gametes, as observed in this study. Earlier reports suggest that bacteria could be intricately involved in the cell death of male gametes as a greater proportion of apoptotic spermatozoa was observed following exposure to pathogens or conditional pathogens [12,49]. Furthermore, sperm cell death was triggered in vitro even by a simple contact with bacteria without the involvement of inflammation [49,50]. Our collected data agree with the above-mentioned studies as we observed a decline in the mitochondrial activity and membrane integrity, accompanied by elevated sperm DNA fragmentation, in the ejaculates obtained from the Lohmann Brown roosters, with a higher occurrence of typical uropathogens.

By nature, the immune system responds to infection by releasing white blood cells to the source of inflammation. On one hand, leukocytes are crucial for the removal of senescent and/or dead germ cells, while on the other hand, their overactivation, triggered by a tight adherence to the spermatozoa, may lead to phagocytosis of even morphologically normal and viable gametes [49,50,51,52]. It has been previously observed that the presence of notably coliform bacteria may lead to a higher incidence of leukocytes in semen, with subsequent damage, particularly to the membranous structures of the spermatozoa, such as the plasma membrane, acrosome and mitochondria. The higher occurrence of leukocytes in the semen samples containing a higher proportion of uropathogenic bacteria in this study corresponds to the earlier reports [6,7,8] that postulate that bacteria alongside leukocytes compromise the lipid symmetry of the sperm membranes, even in otherwise healthy animals. An accompanying event of the active immune response lies in the release of pro-inflammatory cytokines, which may act as spermatotoxins. In addition to promoting oxidative damage to the sperm proteins, lipids and DNA [53], it has been suggested that these molecules participate in the induction of cell death. Within a large and heterogenous family of pro-inflammatory cytokines, TNF-α, a primary molecule released during infection and/or inflammation, may trigger sperm phosphatidylserine translocation and the onset of apoptosis [54,55]. Among the interleukins, IL-1 and IL-6 also seem to mediate the damage to male gametes, which agrees with our observations of their increasing levels being proportional to a diminished semen quality in the presence of uropathogens. Accordingly, their increased levels, as a consequence of a frequent occurrence of bacteria such as *Escherichia coli*, *Staphylococcus aureus* or *Pseudomonas* spp., have been associated with decreased sperm quality, even in stud animals [7,8,56]. Moreover, cytokines have been previously linked to ROS overproduction [7,8,53], mitochondrial dysfunction and a compromised sperm motility [8,57,58], all of which were also revealed by our results.

In addition to inflammation, oxidative stress plays an important role in mediating damage to male gametes. Spermatozoa, leukocytes and aerobic and facultative anaerobic bacteria release ROS as their metabolic by-products. Even anaerobes can synthesize reactive intermediates, particularly through the Fenton and Haber-Weiss reaction, catalyzed by transition metals [59]. ROS are considered to be metabolic by-products in several uropathogenic bacteria, including *Enterococcus faecalis* [12], *Citrobacter* spp. [60], *Escherichia coli* [61] and *Staphylococcus* spp. [62], an elevated load of which may contribute to the progression of oxidative damage to spermatozoa. In addition to elevated ROS levels, our data reveal a notable rise in protein carbonyls and malondialdehyde in the samples collected from the Lohmann Brown roosters, which also contained a higher prevalence of uropathogens. Supraphysiological ROS may attack the lipid bilayer of sperm membranes, which will have an undesirable impact on the semipermeable properties of the sperm surface. Our findings may furthermore support the hypothesis that cell death could also play an essential role in promoting ROS-inflicted sperm DNA fragmentation [63]. Accordingly, an increase in the proportion of spermatozoa with alterations to the membrane integrity and mitochondrial activity correlated with elevated chromatin damage to spermatozoa in the ejaculates which contained more uropathogenic bacteria, and which has also been reported in infertile subjects suffering from urogenital infections [57,64].

Another important outcome of this study was the increased number of bacterial isolates that were resistant to an array of antibiotics used for the antimicrobial susceptibility test, such as ampicillin, chloramphenicol, tetracycline and tobramycin. This observation is in line with the recent evidence indicating a rising tolerance, or even resistance, of particularly uropathogenic bacteria to the antibacterial molecules used to counteract bacteriospermia in animal production. According to Maasjost et al. [26], 89 out of the 145 *Enterococcus* strains isolated from poultry flocks in Germany were tolerant to three or more antibiotics, particularly to tetracycline, lincomycin and gentamycin. While Moawad et al. [27] detected colistin-tolerant and extended-spectrum β-lactamase-producing *Escherichia coli* in healthy broilers in Egypt, Al Azad et al. [65] studied the occurrence of multidrug-resistant *Escherichia coli* from cloacal swabs of broiler chickens in Bangladesh. Recently, coagulase-negative staphylococci were isolated from healthy turkeys by Moawad et al. [27]; while all of the isolates were unaffected by penicillin, ampicillin and tetracycline, their tolerance rates to chloramphenicol, erythromycin and tigecycline oscillated between 87.20 and 97.40%. Richter et al. [66] detected the presence of methicillin- resistant *Staphylococcus aureus* in 90% of turkey flocks in Germany. In the meantime, Lenický et al. [8] studied the antimicrobial susceptibility of bacteria recovered from Big 6 turkey semen. According to the authors, all of the *Staphylococcus lentus* isolates were resistant to chloramphenicol, tigecycline and linezolid. In the case of *Enterococcus faecium*, resistance was detected against imipenem, while ertapenem was shown to be ineffective against *Escherichia coli* and *Vagococcus fluvialis*. Summarizing all of the above evidence, we may speculate that, in addition to the risks associated with the evolution of antibiotic resistance to the animal production and consumers, the presence of bacteria that are resistant to antibiotics may further aggravate damage to the reproductive structures and cells, which will then accelerate inflammation, as indicated by our assessment of selected cytokines. On the other hand, the local immune response may stimulate the secretion of antibacterial proteins that add another layer to the innate defense mechanisms against pathogens. All things considered, the data collected in this study, alongside the documentation gathered from recent studies, strongly advocates for the necessity of modern, fast and cost-effective screening methods able to routinely assess the bacterial profiles of poultry semen alongside their resistance patterns to antibiotics. This strategy could enable a more precise selection of antibiotics and their appropriate doses in diluents and extenders used for poultry sperm processing and storage.

An intriguing line of defense against pathogens represent specific proteins native to the seminal plasma, which exhibit antibacterial properties, and which may play an important role in the activation of the immune response, antigen presentation and leukocyte migration [67,68]. While it has been previously reported that the exposure of spermatozoa to lipopolysaccharide, produced primarily by *Escherichia coli*, increases the expression levels of β-defensin [68,69], in our case, the molecule was found to be decreased in the ejaculates with a high prevalence of *Enterobacteriaceae*. We may hypothesize that once a critical threshold for the bacterial infestation is surpassed, β-defensin is not able to fully prevent or counteract damage to male gametes. This assumption was recently postulated by Duracka et al. [70], who observed high concentrations of the protein in semen containing high load of commensals, while its expression decreased in samples carrying lower concentrations of a much more aggressive *Staphylococcus aureus*. Similarly, higher levels of β-defensin were observed in turkey semen samples of superior quality, in which the molecule was able to maintain the sperm vitality despite the presence of bacteria such as *Bacillus subtilis*, *Empedobacter brevis* and *Staphylococcus chromogenes*. In parallel to β-defensin, cathelicidin has been reported to possess strong antimicrobial activity against various bacteria, fungi and viruses, even at micromolar concentrations [71]. In agreement with our collected data, the associations between an increased sensitivity to infections and a decreased expression of cathelicidin have been previously observed by Brown et al. [72] and Choi et al. [73]. Moreover, research in poultry has shown notable changes in cathelicidin levels following in vitro infection with bacterial endotoxins, suggesting that this protein could become a suitable marker for the detection of bacteriospermia in poultry breeding [74]. Finally, the higher lysozyme concentrations found in the ejaculates with a better quality in our experiments is in line with a previous study that suggested decreased spermatozoa motility in humans could be closely related to low lysozyme levels in semen [75]. Accordingly, higher lysozyme levels were also correlated with an increased proportion of motile and live spermatozoa in turkeys [76] and wild passerines [77]. The exact involvement of antibacterial properties in the process of the immune response is subject to additional research. According to our collected data, we may speculate that the proteins respond in a more effective manner to predominantly non-pathogenic bacteria, even if these are found in higher concentrations in semen. As antibacterial proteins seem to act in unison with other cellular or molecular components of the immune system, we may assume that their increase in the specimens with a lower prevalence of uropathogens may prevent a large-scale inflammatory response and thus keep the cytokines on a stable low level. This is an intriguing finding as, in theory, broiler breeds should present with a diminished immunity. Nevertheless, it seems that the reproductive tract of broiler breeds secretes higher amounts of antibacterial proteins, possibly to add another layer of protection against a potential infection. Whether antibacterial proteins serve as defense molecules exclusively to reproductive fluids or if they act on a more systemic level, is subject to further elucidation. Nevertheless, we may hypothesize that these proteins could be utilized as alternative antibacterial supplements to poultry semen extenders alone or in combination with appropriately selected and dosed antibiotics.

## 4. Materials and Methods

### 4.1. Semen Samples

The semen samples were collected through the cloacal massage of thirty sexually mature Lohmann Brown roosters (representing an egg-laying breed) and thirty ROSS 308 roosters (representing a broiler breed). The animals were 60–65 weeks old and kept at a local poultry breeding farm (Liaharenský podnik Nitra, a.s., Párovské Háje, Slovakia). Shortly before semen collection, the animals were allowed to defecate, and their cloacae were washed with water and soap. Disposable gloves were changed between each semen collection. The ejaculates were collected into sterile collection syringes and immediately transported to the laboratory in the Mini Bio Isotherm vessel, maintaining a constant temperature of 37 °C (M and G Int., Renate, Italy). Each semen specimen was split into three aliquots. The first aliquot was immediately subjected to the assessment of sperm motility, membrane, acrosome and DNA integrity, reactive oxygen species (ROS) production and leukocyte concentration. The second aliquot was transferred to an Eppendorf tube and stored at −80 °C for bacteriological analysis. The third aliquot was centrifuged at 300× *g* for 10 min to obtain the seminal plasma, which was subjected to the assessment of protein concentration and subsequently stored at −80 °C for the evaluation of total antioxidant capacity (TAC) and ELISA assays of proinflammatory markers and antibacterial proteins. The cell pellet was solubilized in RIPA lysis buffer (Merck, Darmstadt, Germany) containing a proteinase inhibitor cocktail (Sig-ma-Aldrich, St. Louis, MO, USA) overnight at 4 °C to allow a complete sperm lysis. The following day, the samples were centrifuged at 13,000× *g* for 30 min, the supernatant was aspirated, subjected to the determination of the protein concentration, and stored at −80 °C for later assessment of oxidative damage to the proteins and lipids. The protein concentration in the seminal plasma and cell lysates was determined using the Total Proteins commercial kit (Waterbury, CT, USA) and RX Monza semi-automated analyzer (Randox, Crumlin, UK) [8].

### 4.2. Bacteriological Analysis

Neat semen samples were subjected to bacteriological analysis according to Lenický et al. [8]. Briefly, 100 μL of each semen specimen were inoculated onto a selection of sterile agars (xylose lysine deoxycholate agar; blood agar base no. 2; soybean casein digest agar; Gassner agar, NutriSelect^®^ basic; Merck, Darmstadt, Germany) and incubated under aerobic conditions at 36 ± 2 °C for 24 h. Bacterial colonies that had grown on the agars were isolated, purified and identified using the Biotyper MALDI-TOF mass spectrometer (Brucker Daltonics, Bremen, Germany) equipped with the Microflex LT instrument and the flexControl software version 3.4. Obtained spectra were processed with the MALDI Biotyper Bruker Taxonomy database (Bruker Daltonics, Bremen, Germany).

### 4.3. Biodiversity Analysis

The absolute number of species recovered from the studied groups was determined as species richness. The standard diversity characteristics were assessed by the BPMSG Diversity Calculator. At the same time, the Berger-Parker Index was calculated following the formula d = max(pi) in order to describe the real unbalanced group differences among the pre-established groups. The distribution of the individual species was graphically compared using MS Excel. ANOVA was calculated for the pre-established groups using the astatsa.com free software platform (version 1.0.1).

### 4.4. Antibiotic Resistance Analysis

Selected bacterial isolates identified in the semen specimens were tested for antibiotic resistance. The disc diffusion method was used to perform the microbial susceptibility test against (10 mg) ampicillin (AMP), chloramphenicol (C), gentamycin (GEN), imipenem (IMP), levofloxacin (LEV), tetracycline (TET), tigecycline (TGC), tobramycin (TOB) according to Kačániová et al. [78].

### 4.5. Conventional Semen Quality Parameters

Sperm motility, expressed as the percentage of spermatozoa moving faster than 5 μm/s, was evaluated with the computer-assisted sperm analysis (CASA) system (version 14.0 TOX IVOS II, Hamilton-Thorne Biosciences, Beverly, CA, USA). Diluted specimens (7 µL) were pipetted to the Makler counting chamber (10 µm depth; Sefi Medical Instruments, Haifa, Israel), which was placed to a pre-heated plate (37 °C) subsequently inserted to the CASA system. The computer system then scanned 10 different microscopic fields within the Makler chamber, thus automatically evaluating the sperm motion activity. The system was set up according to the manufacturer’s instructions for poultry spermatozoa: minimum contrast—50; frame rate—60 Hz; static head intensity—0.22–2.63; static head size—0.16–8.20; static elongation—0–47; default cell intensity—80; default cell size—25 pixels. Sperm motility is expressed as a percentage (%) [8].

Plasma membrane integrity was assessed with the eosin-nigrosin colorimetric methodology. Five μL of each sample were stained with 10 μL eosin (Eosin Y; Sigma-Aldrich, St. Louis, MO, USA) followed by 10 μL nigrosin (Sigma-Aldrich, St. Louis, MO, USA) on a microscopic slide. A smear was prepared using a second slide, which was then allowed to dry at laboratory temperature. The slides were assessed with the Leica DM IL LED inverted microscope (Leica Microsystems, Wetzlar, Germany). Three hundred cells were counted on each microscopic slide by one observer with experience in microscopy, and the proportion of membrane intact spermatozoa is expressed in percentage (%) [79].

The integrity of the acrosomal structures was evaluated with the fast green and rose bengal staining protocol. Each sample (20 μL) was stained with 20 μL of a mixture consisting of both stains (Sigma-Aldrich, St. Louis, MO, USA) and incubated at laboratory temperature for 70 s. Afterwards, 10 μL of the stained sample were smeared on a microscopic slide and air-dried. All slides were evaluated by one observer with experience in microscopy under the Leica DM IL LED microscope by counting 300 cells. Acrosome integrity was expressed as the percentage of cells with an intact acrosomal cap (%) [79].

Mitochondrial activity was assessed with the JC-1 Mitochondrial Membrane Potential Assay (Cayman Chemical, Ann Arbor, MI, USA) employing the JC-1 dye (5.5′,6.6′-tetrachloro-1,1′,3,3′-tetraethylbenzimidazolylcarbocyanine iodide), which was mixed (5 μL) with 100 μL of each specimen. Following incubation (37 °C, 30 min), the samples were centrifuged (150× *g*, 25 °C, 5 min) and washed twice with a washing buffer. Finally, the samples were pipetted into to a dark 96-well plate that was subsequently processed with the combined GloMax Multi^+^ spectro-fluoro-luminometer (Promega, Madison, WI, USA). Mitochondrial membrane potential is expressed as the ratio of JC-1 complexes (green fluorescence) to JC-1 monomers (red fluorescence) [8,79].

Sperm DNA fragmentation was evaluated with the APO-DIRECT^TM^ TUNEL assay kit (BD Biosciences; Franklin Lakes, NJ, USA). One-million sperm cells were fixed in 4% paraformaldehyde (Centralchem, Bratislava, Slovakia), incubated on ice for 1 h, washed 3 times in Dulbecco’s phosphate-buffered saline (DPBS) without calcium and magnesium (Sigma-Aldrich, St. Louis, MO, USA), transferred to 70% ice-cold ethanol (Centralchem, Bratislava, Slovakia) and stored at −20 °C overnight. The next day, the cells were washed, labeled with the DNA labeling solution, rinsed and centrifuged (805× *g*, 5 min) twice. Each sample was then counterstained with DAPI (4′,6-diamidino-2-phenylindole; Sigma-Aldrich, St. Louis, MO, USA; 1 μmol/L in DPBS), pipetted into a dark 96-chamber plate that was processed with the GloMax Multi^+^ spectro-fluoro-luminometer. The proportion of spermatozoa with DNA damage is expressed in percentage (%) [79].

The presence of leukocytes in each semen sample was assessed with the Endtz test. Each sample was stained with the Endtz solution consisting of benzidine (Sigma-Aldrich, St. Louis, MO, USA), 96% ethanol (Centralchem, Bratislava, Slovakia), 3% hydrogen peroxide (H_2_O_2_; Sigma-Aldrich, St. Louis, MO, USA) and sterile water. Following incubation (20 °C, 5 min), the samples were transferred to the Makler chamber and stained round cells were counted with the Nikon ECLIPSE E100 bright-field microscope (Nikon, Tokyo, Japan; ×1000). The results are expressed as ×10^6^ leukocytes/mL semen [8].

### 4.6. Oxidative Profile

Luminol-based chemiluminescent assay was used to quantify the extent of ROS production in the semen specimens. Briefly, each sample was transferred to a 96-well plate and stained with 5 mM luminol working solution (5-amino-2,3-dihydro-1,4-phthalazinedione; Sigma-Aldrich, St. Louis, MO, USA). Negative controls comprised DPBS and luminol, while positive controls were composed of DPBS, H_2_O_2_ (33%; Sigma-Aldrich, St. Louis, MO, USA) and luminol. The light signal produced by the reaction was captured with the GloMax Multi^+^ combined spectro-fluoro-luminometer (Promega Corporation, Madison, WI, USA). The results are expressed in relative light units per second per one-million spermatozoa (RLU/s/10^6^ sperm) [8].

A chemiluminescent protocol introduced by Muller et al. [80] was used to study the TAC of the seminal plasma. Each specimen was mixed with a signal reagent composed of luminol, 4-iodophenol (Sigma-Aldrich; St. Louis, MO, USA), horseradish peroxidase (HRP; Sigma-Aldrich; St. Louis, MO, USA) and H_2_O_2_. The resulting chemiluminescence was monitored during 10 consecutive cycles of 1 min with the GloMax Multi^+^ combined spectro-fluoro-luminometer (Promega Corporation, Madison, WI, USA). The collected results were processed using a standard curve consisting of increasing Trolox concentrations (5–100 μmol/L; 6-hydroxy-2,5,7,8-tetramethylchroman-2-carboxylic acid; Sigma-Aldrich; St. Louis, MO, USA). The results are expressed as μmol Trolox Eq./g protein [8].

Protein oxidation expressed as the concentration of protein carbonyls (PC) in the sperm lysates was evaluated with a modified DNPH (dinitrophenylhydrazine) assay [81]. Each sperm lysate was adjusted with distilled water to 1 mg protein/1 mL, pre-treated with 1 mL of trichloroacetic acid (TCA; Sigma-Aldrich, St. Louis, MO, USA) and incubated for 10 min at 4 °C. Following centrifugation (805× *g*, 10 min, 4 °C) the pellet was mixed with 1 mL DNPH (Sigma-Aldrich, St. Louis, MO, USA) and incubated at 37 °C for 1 h. Following incubation, 1 mL TCA was added to the samples, which were then cooled down and centrifuged again (805× *g*, 5 min, 4 °C). The pellet was washed 3 times with 500 µL ethyl acetate/ethanol (50/50 mix; Sigma-Aldrich, St. Louis, MO, USA). Finally, the pellet was mixed with 1 mL 6 M guanidine hydrochloride (Sigma-Aldrich, St. Louis, MO, USA) and absorbance of the mixture was measured at 360 nm using a Cary UV-VIS spectrophotometer (Cary Systems, Santa Clara, CA, USA). Protein oxidation is expressed in nmol PC/mg protein [8].

The extent of lipid peroxidation expressed through malondialdehyde (MDA) levels was quantified with the thiobarbituric acid-reactive substances (TBARS) assay. Briefly, the sperm lysates were pre-treated with 5% SDS (sodium dodecyl sulfate; Sigma-Aldrich, St. Louis, MO, USA) and subsequently mixed with 0.53% thiobarbituric acid (Sigma-Aldrich, St. Louis, MO, USA), dissolved in 20% acetic acid (pH 3.5; Centralchem, Slovakia). The samples were boiled (100 °C) for 1 h, afterwards cooled down on ice for 10 min and centrifuged (1300× *g*, 10 min, 4 °C). The obtained supernatants were transferred to a 96-well plate, and absorbances were measured with the GloMax plate spectrophotometer (Promega Corporation, Madison, WI, USA) at 540 nm. The collected results were processed using a standard curve constructed from the commercially available MDA standards (Cayman Chemical; Ann Arbor, MI, USA). Oxidative damage to lipids is expressed in µmol MDA/mg protein [8].

### 4.7. ELISA Assays

Commercially available ELISA kits designed for samples of chicken origin were purchased from MyBioSource (San Diego, CA, USA) for the quantification of selected pro-inflammatory factors including tumor necrosis factor alpha (TNF-α; Cat. # MBS746318), interleukin-1 beta (IL-1β; Cat. # MBS454453), interleukin-6 (IL-6; Cat. # MBS2021018) and C-reactive protein (CRP; Cat. # MBS764341), as well as selected proteins with antibacterial properties comprising cathelicidin (Cat. # MBS735193), beta-defensin (Cat. # MBS018020) and lysozyme (Cat. # MBS701562). A double-sandwich ELISA protocol was performed according to the instructions of the manufacturer and the absorbances were read with the help of the GloMax plate spectrophotometer (Promega, Madison, WI, USA) at 450 nm.

### 4.8. Statistics

The collected data were statistically processed with the GraphPad Prism program (version 9.4.1 for Mac; GraphPad Software Incorporated, La Jolla, CA, USA). The results are displayed as mean (±SD). The Shapiro-Wilk normality test followed by the Mann-Whitney U nonparametric test were selected for statistical analysis. Differences were considered as significant (*) if *p* < 0.05.

## 5. Conclusions

As poultry production directly depends upon the quality of rooster ejaculates for artificial insemination, attention must be paid to all the factors that potentially endanger sperm structural integrity and functional activity during semen collection, processing, and storage. The data collected from our experiments revealed that bacteria are present even in ejaculates of rooster studs kept for insemination purposes. We observed that the bacterial profiles were unique to each breed, and the prevalence of uropathogens had a more decisive impact on the resulting sperm quality, as opposed to the general bacterial load. In addition to oxidative stress and inflammation, as important hallmarks of bacteriospermia, antibacterial proteins native to poultry semen seem to play important roles in the prevention of bacteria-inflicted damage to male gametes and could be used as supplements to poultry semen extenders in the future. Finally, a substantial proportion of the bacterial isolates recovered from the semen presented with antibiotic resistance, which fortifies the need for a more vigorous bacteriological screening of semen samples used for reproductive technologies, as well as for the development of strategies to prevent the spread of bacterial resistance in poultry industry.

## Figures and Tables

**Table 1:** Bacterial profiles of semen samples collected from Lohmann Brown and ROSS 308 roosters.

Groups	Lohmann Brown (n = 30)	ROSS 308 (n = 30)
Bacterial Load (log_10_ CFU/mL)	7.23 ± 0.64	13.44 ± 1.97 *
Identified Bacterial Species and Sample Positivity	*Escherichia coli* (93.00%)	*Staphylococcus epidermidis* (30.00%)
	*Enterococcus faecalis* (50.00%)	*Lactobacillus johnsonii* (23.30%)
	*Citrobacter braakii* (33.30%)	*Escherichia coli* (20.00%)
	*Enterococcus casseliflavus* (16.70%)	*Citrobacter braakii* (16.70%)
	*Pseudomonas putida* (17.70%)	*Enterococcus faecalis* (13.30%)
	*Corynebacterium glutamicum* (13.30%)	*Micrococcus luteus* (13.30%)
	*Enterococcus avium* (10.00%)	*Enterobacter cloacae* (10.00%)
	*Serratia liquefaciens* (10.00%)	*Enterococcus casseliflavus* (10.00%)
	*Acinetobacter baumannii* (6.70%)	*Ochrobactrum intermedium* (10.00%)
	*Alcaligenes faecalis* (6.70%)	*Oligella urethralis* (3.33%)
	*Ochrobactrum anthropi* (6.70%)	*Acinetobacter guillouiae* (3.33%)
	*Pseudomonas aeruginosa* (6.70%)	*Bacillus oceanisediminis* (3.33%)
	*Pseudomonas pseudoalcaligenes* (6.70%)	*Glutamicibacter creatinolyticus* (3.33%)
	*Rothia terrae* (6.70%)	*Lelliottia amnigena* (3.33%)
	*Staphylococcus aureus* (6.70%)	*Staphylococcus saprophyticus* (3.33%)
	*Streptococcus pluranimalium* (6.70%)	*Streptococcus pluranimalium* (3.33%)
	*Corynebacterium xerosis* (3.33%)	
	*Klebsiella oxytoca* (3.33%)	
	*Macrococcus caseolyticus* (3.33%)	
	*Pantoea agglomerans* (3.33%)	
	*Pseudomonas composti* (3.33%)	
	*Staphylococcus hyicus* (3.33%)	
	*Staphylococcus warneri* (3.33%)	

* *p* < 0.05.

**Table 2:** Bacterial biodiversity characteristics of the analyzed breeds.

Groups	Lohman Brown	ROSS 308
Richness (R)	23	16
Berger Parker Dominance Index	0.29	0.18
Shannon α-diversity	0.03	0.02
Simpson dominance	0.14	0.1

**Table 3:** Resistance profiles of bacteria recovered from Lohmann Brown (LB) and ROSS 308 (R 308) rooster semen.

Bacterium	Sensitivity	AMP	GEN	C	TET	IMP	TOB	TGC	LEV
*Acinetobacter baumannii*	S	100%	50%	100%	100%	100%	50%	100%	100%
	I	0%	50%	0%	0%	0%	50%	0%	0%
	R	0%	0%	0%	0%	0%	0%	0%	0%
*Acinetobacter guillouiae*	S	0%	100%	100%	100%	100%	100%	100%	100%
	I	100%	0%	0%	0%	0%	0%	0%	0%
	R	0%	0%	0%	0%	0%	0%	0%	0%
*Alcaligenes faecalis*	S	100%	0%	100%	0%	100%	100%	100%	100%
	I	0%	100%	0%	100%	0%	0%	0%	0%
	R	0%	0%	0%	0%	0%	0%	0%	0%
*Bacillus oceanisediminis*	S	0%	100%	ND	0%	100%	ND	100%	100%
	I	100%	0%		100%	0%		0%	0%
	R	0%	0%		0%	0%		0%	0%
*Citrobacter braakii*	S	0%	100%	66%	100%	100%	33%	100%	83%
	I	25%	0%	17%	0%	0%	50%	0%	17%
	R	75%	0%	17%	0%	0%	17%	0%	0%
*Corynebacterium glutamicum*	S	100%	100%	ND	100%	100%	ND	100%	100%
	I	0%	0%		0%	0%		0%	0%
	R	0%	0%		0%	0%		0%	0%
*Corynebacterium xerosis*	S	0%	100%	100%	100%	100%	100%	100%	100%
	I	100%	0%	0%	0%	0%	0%	0%	0%
	R	0%	0%	0%	0%	0%	0%	0%	0%
*Enterobacter cloacae*	S	0%	100%	100%	100%	100%	50%	100%	100%
	I	50%	0%	0%	0%	0%	50%	0%	0%
	R	50%	0%	0%	0%	0%	0%	0%	0%
*Enterococcus casseliflavus*	S	40%	100%	100%	100%	100%	100%	100%	100%
	I	60%	0%	0%	0%	0%	0%	0%	0%
	R	0%	0%	0%	0%	0%	0%	0%	0%
*Enterococcus avium*	S	50%	50%	100%	100%	100%	100%	100%	100%
	I	50%	50%	0%	0%	0%	0%	0%	0%
	R	0%	0%	0%	0%	0%	0%	0%	0%
*Enterococcus faecalis*	S	50%	100%	100%	100%	75%	100%	75%	100%
	I	25%	0%	0%	0%	0%	0%	0%	0%
	R	25%	0%	0%	0%	25%	0%	25%	0%
*Escherichia coli*	S	18%	100%	64%	64%	100%	82%	100%	100%
	I	36%	0%	0%	36%	0%	0%	0%	0%
	R	46%	0%	36%	0%	0%	18%	0%	0%
*Glutamicibacter creatinolyticus*	S	100%	100%	100%	100%	100%	100%	100%	100%
	I	0%	0%	0%	0%	0%	0%	0%	0%
	R	0%	0%	0%	0%	0%	0%	0%	0%
*Klebsiella oxytoca*	S	100%	0%	100%	100%	100%	0%	100%	100%
	I	0%	100%	0%	0%	0%	0%	0%	0%
	R	0%	0%	0%	0%	0%	100%	0%	0%
*Lactobacillus johnsonii*	S	50%	100%	ND	33.3%	100%	100%	100%	100%
	I	50%	0%		33.3%	0%	0%	0%	0%
	R	0%	0%		33.3%	0%	0%	0%	0%
*Lelliottia amnigena*	S	67%	67%	100%	100%	100%	100%	100%	100%
	I	33%	33%	0%	0%	0%	0%	0%	0%
	R	0%	0%	0%	0%	0%	0%	0%	0%
*Macrococcus caseolyticus*	S	100%	100%	100%	100%	100%	100%	100%	100%
	I	0%	0%	0%	0%	0%	0%	0%	0%
	R	0%	0%	0%	0%	0%	0%	0%	0%
*Micrococcus luteus*	S	100%	100%	ND	100%	100%	ND	100%	100%
	I	0%	0%		0%	0%		0%	0%
	R	0%	0%		0%	0%		0%	0%
*Ochrobactrum anthropi*	S	0%	100%	ND	100%	100%	ND	100%	100%
	I	0%	0%		0%	0%		0%	0%
	R	100%	0%		0%	0%		0%	0%
*Ochrobactrum intermedium*	S	0%	100%	ND	100%	100%	ND	100%	100%
	I	50%	0%		0%	0%		0%	0%
	R	50%	0%		0%	0%		0%	0%
*Oligella urethralis*	S	100%	100%	100%	100%	100%	100%	100%	0%
	I	0%	0%	0%	0%	0%	0%	0%	100%
	R	0%	0%	0%	0%	0%	0%	0%	0%
*Pantoea agglomerans*	S	0%	100%	100%	100%	100%	100%	100%	100%
	I	100%	0%	0%	0%	0%	0%	0%	0%
	R	0%	0%	0%	0%	0%	0%	0%	0%
*Pseudomonas aeruginosa*	S	100%	100%	100%	100%	100%	100%	100%	100%
	I	0%	0%	0%	0%	0%	0%	0%	0%
	R	0%	0%	0%	0%	0%	0%	0%	0%
*Pseudomonas composti*	S	100%	100%	100%	100%	100%	100%	100%	100%
	I	0%	0%	0%	0%	0%	0%	0%	0%
	R	0%	0%	0%	0%	0%	0%	0%	0%
*Pseudomonas pseudoalcaligenes*	S	100%	100%	100%	100%	100%	100%	100%	100%
	I	0%	0%	0%	0%	0%	0%	0%	0%
	R	0%	0%	0%	0%	0%	0%	0%	0%
*Pseudomonas putida*	S	33%	67%	100%	100%	33%	100%	100%	100%
	I	67%	33%	0%	0%	67%	0%	0%	0%
	R	0%	0%	0%	0%	0%	0%	0%	0%
*Rothia terrae*	S	100%	0%	100%	100%	100%	100%	100%	100%
	I	0%	100%	0%	0%	0%	0%	0%	0%
	R	0%	0%	0%	0%	0%	0%	0%	0%
*Serratia liquefaciens*	S	0%	100%	100%	100%	100%	100%	100%	100%
	I	0%	0%	0%	0%	0%	0%	0%	0%
	R	100%	0%	0%	0%	0%	0%	0%	0%
*Staphylococcus aureus*	S	100%	100%	100%	0%	100%	100%	100%	100%
	I	0%	0%	0%	100%	0%	0%	0%	0%
	R	0%	0%	0%	0%	0%	0%	0%	0%
*Staphylococcus epidermidis*	S	40%	100%	100%	20%	100%	20%	100%	100%
	I	40%	0%	0%	20%	0%	40%	0%	0%
	R	20%	0%	0%	60%	0%	40%	0%	0%
*Staphylococcus hyicus*	S	0%	100%	100%	0%	100%	100%	100%	0%
	I	100%	0%	0%	100%	0%	0%	0%	100%
	R	0%	0%	0%	0%	0%	0%	0%	0%
*Staphylococcus saprophyticus*	S	0%	100%	100%	100%	100%	100%	100%	0%
	I	100%	0%	0%	0%	0%	0%	0%	100%
	R	0%	0%	0%	0%	0%	0%	0%	0%
*Staphylococcus warneri*	S	0%	100%	100%	100%	100%	0%	100%	100%
	I	100%	0%	0%	0%	0%	0%	0%	0%
	R	0%	0%	0%	0%	0%	100%	0%	0%
*Streptococcus pluranimalium*	S	100%	100%	100%	100%	100%	100%	100%	100%
	I	0%	0%	0%	0%	0%	0%	0%	0%
	R	0%	0%	0%	0%	0%	0%	0%	0%

AMP—ampicillin, C—chloramphenicol, GEN—gentamycin, IMP—imipenem, LEV—levofloxacin, TET—tetracycline, TGC—tigecycline, TOB—tobramycin. ND—not defined, S—sensitive, R—resistant.

## Data Availability

The data presented in this study are available on request from the corresponding author.
